# New Coleoptera records from New Brunswick, Canada: Cerambycidae

**DOI:** 10.3897/zookeys.179.2601

**Published:** 2012-04-04

**Authors:** Reginald P. Webster, Jon D. Sweeney, Ian DeMerchant, Peter J. Silk, Peter Mayo

**Affiliations:** 1 Natural Resources Canada, Canadian Forest Service - Atlantic Forestry Centre, 1350 Regent St., P.O. Box 4000, Fredericton, NB, Canada E3B 5P7

**Keywords:** Cerambycidae, new records, Canada, New Brunswick

## Abstract

Five species of Cerambycidae, *Acmaeops discoideus* (Haldeman), *Anelaphus villosus* (Fabricius), *Phymatodes* species (CNC sp. n. #1), *Sarosesthes fulminans* (Fabricius), and *Urgleptus signatus* (LeConte) are newly recorded for New Brunswick, Canada. All but *Acmaeops villosus* are new to the Maritime provinces. *Phymatodes testaceus* (Linnaeus) is removed from the faunal list of the province as a result of mislabeled specimens, records of *Phymatodes maculicollis* LeConte are presented confirming the presence of this species in New Brunswick, and the first recent records of*Neospondylis upiformis* (Mannerheim) are presented. Additional records are given for the recently recorded *Phymatodes aereus* (Newman), indicating a wider distribution in the province. Collection data, habitat data, and distribution maps are presented for each species.

## Introduction

The Cerambycidae (longhorn beetles) fauna of New Brunswick and the Maritime provinces (New Brunswick, Nova Scotia, Prince Edward Island) were reviewed by McCorquodale and Bondrup-Nielsen (2004) and Webster et al. (2009). Majka et al. (2007) reviewed the fauna of Prince Edward Island. Later, McCorquodale (2010) reviewed the longhorn beetle fauna of the Atlantic Maritime Ecozone in the context of collections and sources of information, the geographic distribution within the ecozone and overall global distribution, host plant usage, and anthropogenic effects on the fauna. Webster et al. (2009) reported 48 species as new to the province, bringing the total number of species known from New Brunswick to 116. More recently, Majka et al. (2010) added *Phymatodes aereus* (Newman) and *Typocerus sparsus* LeConte to the faunal list. Here, we add six species of Cerambycidae to the faunal list of New Brunswick.

## Methods and conventions

### Collection methods

Most specimens were collected from Lindgren funnel trap samples during a study to develop a general attractant for the detection of invasive species of Cerambycidae. These traps mimic tree trunks and are often effective for sampling species of Coleoptera that live in microhabitats associated with standing trees (Lindgren 1983). [See Webster et al. (in press) for details of the methods used to deploy Lindgren 12-funnel traps and for sample collection.] Traps were baited with commercially purchased high-release-rate lures of ethanol, α-pinene (mixture of 93% (-) and 7% (+) α-pinene), or “BSLB kairomone” (Contech International, Inc.; a blend of monoterpenes attractive to *Tetropium fuscum* (Fabr.) and *Tetropium cinnamopterum* Kirby) (Sweeney et al. 2006) or with racemic 3-hydroxyhexan-2-one or racemic 3-hydroxyoctan-2-one, synthesized at the Fredericton laboratory (See Hanks et al. (2007) and reference therein for details on their synthesis) and loaded into release devices by Contech International, Inc. (Delta BC). Specific enantiomers of the latter compounds have been identified as long-distance sex/aggregation pheromones in several species of Cerambycinae (Fettköther et al. 1995; Leal et al. 1995; Lacey et al. 2004, 2007). The pheromones are naturally emitted by males and attract females or both sexes depending on the species (Lacey et al. 2004, 2007; Hanks et al. 2007). A description of the habitat was recorded for all specimens collected during this survey. Locality and habitat data are presented exactly as on labels for each record. This information, as well as additional collecting notes, is summarized and discussed in the collection and habitat data section for each species.

## Distribution

Distribution maps, created using ArcMap and ArcGIS, are presented for each species in New Brunswick. Every species is cited with current distribution in Canada and Alaska, using abbreviations for the state, provinces, and territories. New records for New Brunswick are indicated in bold under Distribution in Canada and Alaska. The following abbreviations are used in the text:

Acronyms of collections examined or where specimens reside referred to in this study are as follows:

**AFC** Atlantic Forestry Centre, Natural Resources Canada, Canadian Forest Service, Fredericton, New Brunswick, Canada

**CNC** Canadian National Collection of Insects, Arachnids and Nematodes, Agriculture and Agri-Food Canada, Ottawa, Ontario, Canada

**NBM** New Brunswick Museum, Saint John, New Brunswick, Canada

**RWC** Reginald Webster Collection, Charters Settlement, New Brunswick, Canada

**Table:** 

**AK**	Alaska	**MB**	Manitoba
**YT**	Yukon Territory	**ON**	Ontario
**NT**	Northwest Territories	**QC**	Quebec
**NU**	Nunavut	**NB**	New Brunswick
**BC**	British Columbia	**PE**	Prince Edward Island
**AB**	Alberta	**NS**	Nova Scotia
**SK**	Saskatchewan	**NF & LB**	Newfoundland and Labrador*

* Newfoundland and Labrador are each treated separately under the current Distribution in Canada and Alaska.

## Results

### Species accounts

All records below are species newly recorded for New Brunswick, Canada, unless noted otherwise (additional records). Species followed by ** are newly recorded from the Maritime provinces of Canada.

#### Family Cerambycidae Latreille, 1802. Subfamily Spondylidinae Audinet-Serville, 1832. Tribe Spondylidini Audinet-Serville, 1832

##### Neospondylis upiformis

(Mannerheim, 1843)

http://species-id.net/wiki/Neospondylis_upiformis

Map 1

###### Material examined.

**Additional New Brunswick records, Restigouche Co.**, Dionne Brook P.N.A. (Protected Natural Area), 47.9064°N, 68.3441°W, 31.V–15.VI.2011, 27.VI–14.VII.2011, M. Roy & V. Webster, old-growth white spruce and balsam fir forest, Lindgren funnel traps (5, AFC, NBM, RWC).

**Collection and habitat data.** Adults were captured in Lindgren funnel traps deployed in an old-growth white spruce (*Picea glauca* (Moench) Voss) and balsam fir (*Abies balsamea* (L.) Mill.) forest. Specimens were captured during June and July in New Brunswick. Larvae of this species probably feed in the roots of fir (*Abies*) or pine (*Pinus*) (Yanega 1996).

**Distribution in Canada and Alaska.** AK, BC, AB, ON, QC, NB, NS, NF (McNamara 1991; Smith and Hurley 2006; Webster et al. 2009; Majka and Ogden 2010). Webster et al. (2009) newly reported this species from New Brunswick based on a specimen collected by Charles E. Atwood in Boiestown, Northumberland Co. (specimen is in the Royal Ontario Museum, Toronto). No date was given on the label, but Charles Atwood made a few collecting trips to New Brunswick during the 1930s (McCorquodale 2010). The record from the Dionne Brook P.N.A. is the first recent record of this species from the province.

**Map 1.**
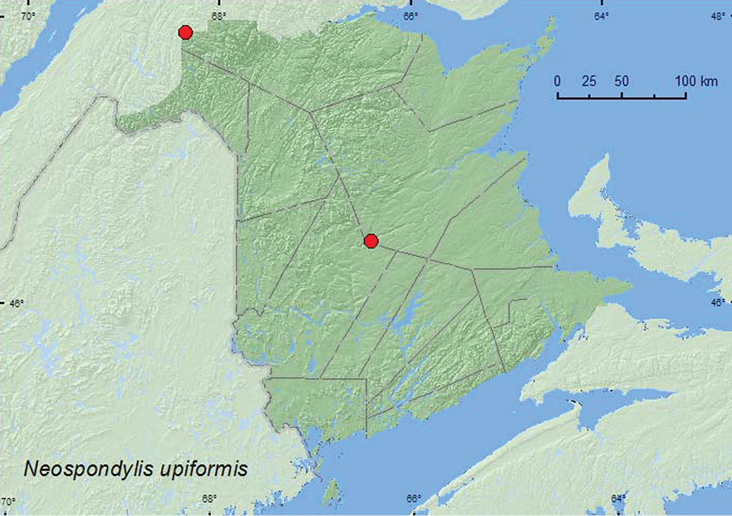
Collection localities in New Brunswick, Canada of *Neospondylis upiformis*.

##### Subfamily Lepturinae Latreille, 1802. Tribe Lepturini Latreille, 1802

###### Acmaeops discoideus

(Haldeman, 1847)

http://species-id.net/wiki/Acmaeops_discoideus

Map 2

####### Material examined.

**New Brunswick, York Co.**, 8.1 km W of Tracy off Rt. 645, 45.6880°N, 66.7841°W, 30.VI.2010, R. P. Webster, roadside, on *Sambucus canadensis* flowers (1, RWC).

####### Collection and habitat data.

 One individual was collected in late June from flowers of elderberry (*Sambucus canadensis* L.) on a roadside near an area with red pine (*Pinus resinosa* Ait.) and white pine (*Pinus strobus* L.). The larvae of this species develop in *Pinus* spp. (Yanega 1996).

**Distribution in Canada and Alaska.** **NB**, NS (Webster et al. 2009). Webster et al. (2009) newly recorded this species from Canada on the basis of a specimen collected in Halifax, Nova Scotia.

**Map 2.**
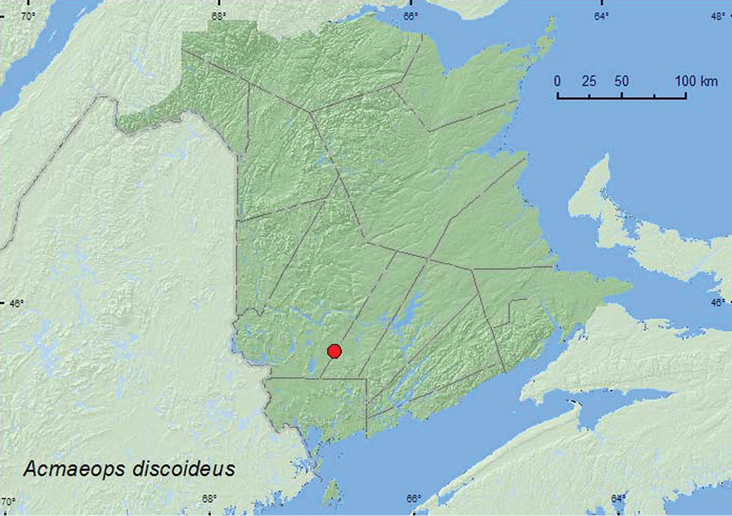
Collection localities in New Brunswick, Canada of *Acmaeops discoideus*.

##### Subfamily Cerambycinae Latreille, 1802. Tribe Callidiini Kirby, 1837

###### Phymatodes aereus

(Newman, 1838)

http://species-id.net/wiki/Phymatodes_aereus

Map 3

####### Material examined.

**Additional New Brunswick records. Queens Co.**, Cranberry Lake P.N.A, 46.1125°N, 65.6075°W, 27.V–5.VI.2009, 11–18.VI.2009, 18–25.VI.2009, 25.VI–1.VII.2009, 7–14.VIII.2009, R. Webster & M.-A. Giguère, old red oak forest, Lindgren funnel traps (37, AFC, RWC); same locality and forest type, 22–29.VI.2011, 29.VI–7.VII.2011, M. Roy & V. Webster, old red oak forest, Lindgren funnel traps (4, AFC, NBM); Grand Lake Meadows P.N.A., 45.8227°N, 66.1209°W, 31.V–15.VI.2009, 15–29.VI.2009, R. Webster & C. MacKay, old silver maple forest with green ash and seasonally flooded marsh, Lindgren funnel traps (10, AFC); same locality data and forest type, 29.VI-12.VII.2009, R. Webster, C. MacKay, M. Laity, & R. Johns, Lindgren funnel trap (1, AFC). **York Co.**, 15 km W of Tracy off Rt. 645, 45.6848°N, 66.8821°W, 7–14.VII.2009, R. Webster & M.-A. Giguère, old red pine forest, Lindgren funnel traps (1, RWC).

####### Collection and habitat data.

*Phymatodes aereus* was captured in Lindgren funnel traps baited with racemic 3-hydroxyhexan-2-one deployed in an old red oak (*Quercus rubra* L.) forest, an old silver maple (*Acer saccharinum* L.) forest, and in an old red pine forest with scattered hardwoods. Larvae of this uncommon species develop in dead *Quercus* (Lingafelter 2007). Adults in New Brunswick were captured during May, June, and July.

####### Distribution in Canada and Alaska.

 ON, QC, NB (McNamara 1991; Majka et al. 2010). Majka et al. (2010) newly recorded this species from New Brunswick and the Maritime provinces, based on a specimen collected by Martin Turgeon in Saint-Basile, Madawaska Co. during 2003. This species is probably widespread in the province.

**Map 3.**
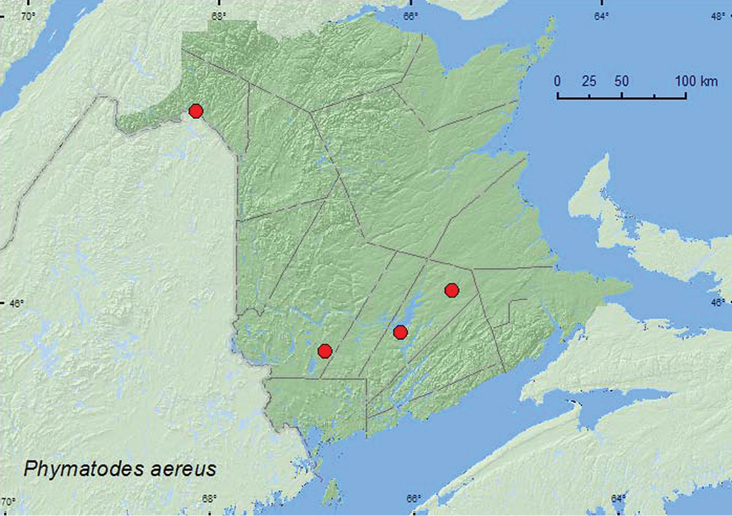
Collection localities in New Brunswick, Canada of *Phymatodes aereus*.

###### Phymatodes maculicollis

LeConte, 1878

http://species-id.net/wiki/Phymatodes_maculicollis

Map 4

####### Material examined.

**Additional New Brunswick records. Charlotte Co.**, 10 km NW of New River Beach, 45.2110°N, 66.6170°W, 15–29.VI.2010, R. Webster & C. MacKay, old growth eastern white cedar forest, Lindgren funnel trap (1, AFC). **Queens Co.**, Cranberry Lake P.N.A, 46.1125°N, 65.6075°W, 27.V-5.VI.2009, 18–25.VI.2009, R. Webster & M.-A. Giguère, old red oak forest, Lindgren funnel trap (2, AFC, RWC). **Restigouche Co.**, Dionne Brook P.N.A., 47.9064°N, 68.3441°W, 27.VI-14.VII.2011, 14–28.VII.2011, M. Roy & V. Webster, old-growth white spruce and balsam fir forest, Lindgren funnel traps (6, AFC, NBM, RWC). **York Co.**, 15 km W of Tracy off Rt. 645, 45.6848°N, 66.8821°W, 21–28.VI.2009, R. Webster & M.-A. Giguère, old red pine forest, Lindgren funnel traps (1, RWC); 14 km WSW of Tracy, S of Rt. 645, 45.6741°N, 66.8661°W, 10–26.V.2010, 2–16.VI.2011, R. Webster & C. MacKay, old mixed forest with red and white spruce, red and white pine, balsam fir, eastern white cedar, red maple, and *Populus* sp., Lindgren funnel trap (14, AFC, RWC).

####### Collection and habitat data.

*Phymatodes maculicollis* larvae develop under bark of spruce (*Picea*) and fir branches (Yanega 1996). This species, which is considered rare in the East, was captured in Lindgren funnel traps baited with racemic 3-hydroxyhexan-2-one deployed in an old-growth eastern white cedar (*Thuja occidentalis* L.) forest, an old-growth white spruce and balsam fir forest, an old red pine forest, an old mixed forest, and in an old red oak forest with scattered conifers. Adults were captured during May, June, and July.

####### Distribution in Canada and Alaska.

 YK, BC, QC, NB (McNamara 1991). McNamara (1991) listed *Phymatodes maculicollis* for New Brunswick but no supporting voucher specimens or other published records could be located by Webster et al. (2009). The above records establish the presence of this species for the province.

**Map 4.**
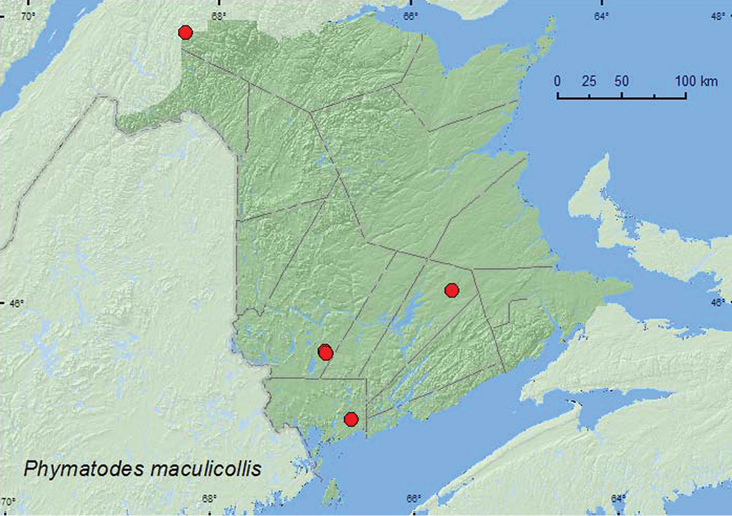
Collection localities in New Brunswick, Canada of *Phymatodes maculicollis*.

###### Phymatodes species (CNC sp. n. #1)**

Map 5

####### Material examined.

**New Brunswick, York Co.**, (Fredericton) Odell Park, 2.VII.2002, (G. Smith) Trap Lindgren Funnel, Lure Ipslure (1, AFC).

####### Collection and habitat data.

One individual was captured during July in a Lindgren funnel trap in an old-growth forest with hemlock (*Tsuga canadensis* (L.) Carr.), American beech (*Fagus grandifolia* Ehrh.), and sugar maple (*Acer saccharum* Marsh.).

####### Distribution in Canada and Alaska.

QC, **NB** (Yanega 1996). According to Yanega (1996)this undescribed species is related to *Phymatodes ater* LeConte but lacks the prominent pronotal calli and possesses finer elytral punctures.

**Map 5.**
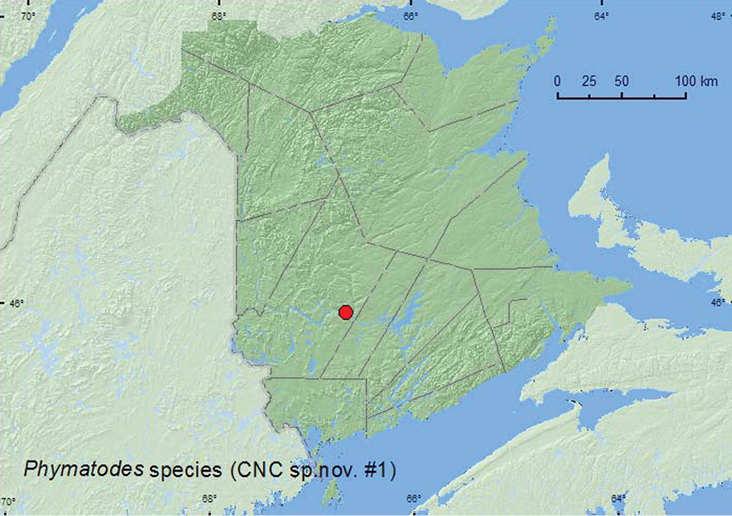
Collection localities in New Brunswick, Canada of *Phymatodes* species (CNC sp. n. #1).

###### Phymatodes testaceus

(Linnaeus, 1758)

http://species-id.net/wiki/Phymatodes_testaceus

Webster et al. (2009) reported *Phymatodes testaceus* from New Brunswick based on a series of specimens collected from Pleasantfield in Queens Co. There are no towns with this name in Queens Co., New Brunswick. The original Forest Insect and Disease Survey slip (at AFC) was examined and Pleasantfield is actually in Queens Co., Nova Scotia, indicating that these specimens had been mislabeled. *Phymatodes testaceus* is accordingly removed from the faunal list of New Brunswick.

###### Tribe Clytini Mulsant, 1839

####### Sarosesthes fulminans

(Fabricius, 1775)**

http://species-id.net/wiki/Sarosesthes_fulminans

Map 6

######## Material examined.

**New Brunswick, Queens Co.**, Cranberry Lake P.N.A, 46.1125°N, 65.6075°W, 11–18.VI.2009, 18–25.VI.2009, 25.VI–1.VII.2009, 15–21.VII.2009, 19.VIII-2.IX.2009, R. Webster & M.-A. Giguère, old red oak forest, Lindgren funnel traps (59, AFC, NBM, RWC); same locality and forest type, 29.VI–7.VII.2011, 20.VII–4.VIII.2011, M. Roy & V. Webster, old red oak forest, Lindgren funnel traps (4, AFC, NBM).

######## Collection and habitat data.

All *Sarosesthes fulminans* specimens but one were captured in Lindgren funnel traps baited with racemic 3-hydroxyhexan-2-one. Lacey et al. (2009) showed that males of this species emit (*R*)-3-hydroxyhexan-2-one and (*2S*, 3*R*)-2,3-hexane-diol and that both sexes were attracted to males in laboratory olfactometer bioassays. Adults were collected in an old red oak forest during June, July, and August. Larvae of this uncommon species occur under bark and in sapwood of oak and other hardwood species (Yanega 1996; Lingafelter 2007).

######## Distribution in Canada and Alaska.

 ON, QC, **NB** (McNamara 1991). This species was abundant at the Cranberry Lake P.N.A. (Protected Natural Area) and will probably be found at other localities in New Brunswick and the other Maritime provinces where red oak occurs using traps baited with racemic 3-hydroxyhexan-2-one.

**Map 6.**
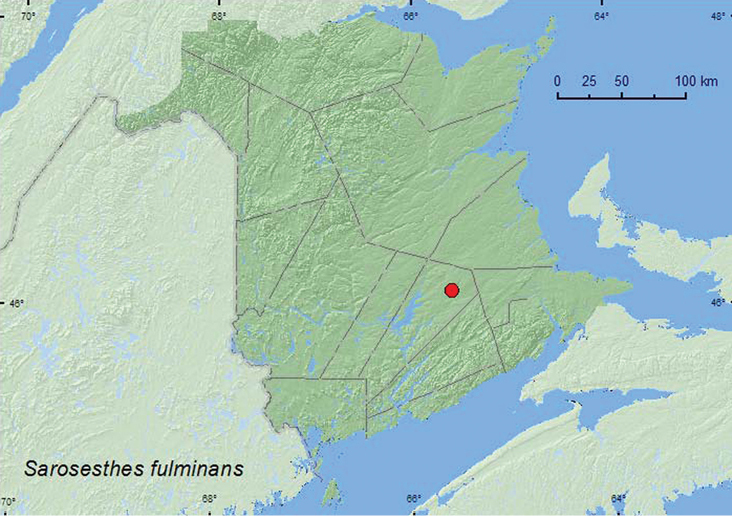
Collection localities in New Brunswick, Canada of *Sarosesthes fulminans*.

###### Tribe Elaphidiini Thomson, 1864

####### Anelaphus villosus

(Fabricius, 1792)

http://species-id.net/wiki/Anelaphus_villosus

Map 7

######## Material examined.

**New Brunswick, Queens Co.**, Cranberry Lake P.N.A, 46.1125°N, 65.6075°W, 29.VI–7.VII.2011, 13–20.VII.2011, 20.VII–4.VIII.2011, 4–18.VIII.2011, M. Roy & V. Webster, old red oak forest, Lindgren funnel traps in forest canopy (7, AFC, NBM, RWC).

######## Collection and habitat data.

Adults were captured during July and August in Lindgren funnel traps baited with ethanol deployed in the forest canopy of an old red oak forest. Larvae are twig pruners of most eastern hardwoods and shrubs (Lingafelter 2007).

######## Distribution in Canada and Alaska.

MB, ON, QC, **NB**, NS (McNamara 1991; McCorquodale and Bondrup-Nielsen 2004).

**Map 7.**
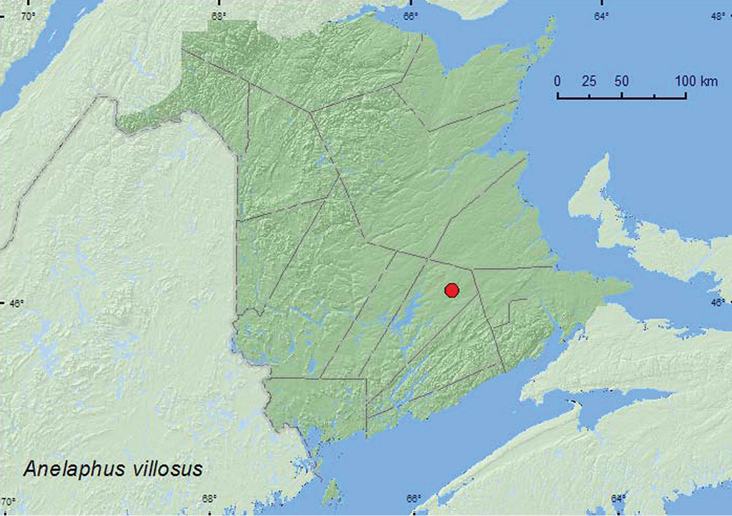
Collection localities in New Brunswick, Canada of *Anelaphus villosus*.

##### Subfamily Lamiinae Latreille, 1825. Tribe Acanthocinini Blanchard, 1845

###### Urgleptus signatus

(LeConte, 1852)**

http://species-id.net/wiki/Urgleptus_signatus

Map 8

####### Material examined. 

**New Brunswick, Queens Co.**, Cranberry Lake P.N.A, 46.1125°N, 65.6075°W, 29.VI–7.VII.2011, 13–20.VII.2011, 20.VII–4.VIII.2011, M. Roy & V. Webster, old red oak forest, Lindgren funnel traps in forest canopy (3, NBM, RWC); same locality data and forest type, 31.VIII–15.IX.2011, R. P. Webster & Cory Hughes, Lindgren funnel trap in forest canopy (1, AFC).

####### Collection and habitat data.

This species was captured in Lindgren funnel traps baited with ethanol deployed in the canopy of an old red oak forest. Adults were collected during July, August, and September. Larvae live in branches of various hardwoods, such as *Acer*, *Cornus*, *Quercus*, and *Tilia* (Lingafelter 2007).

####### Distribution in Canada and Alaska.

 ON, QC, **NB** (McNamara 1991).

**Map 8.**
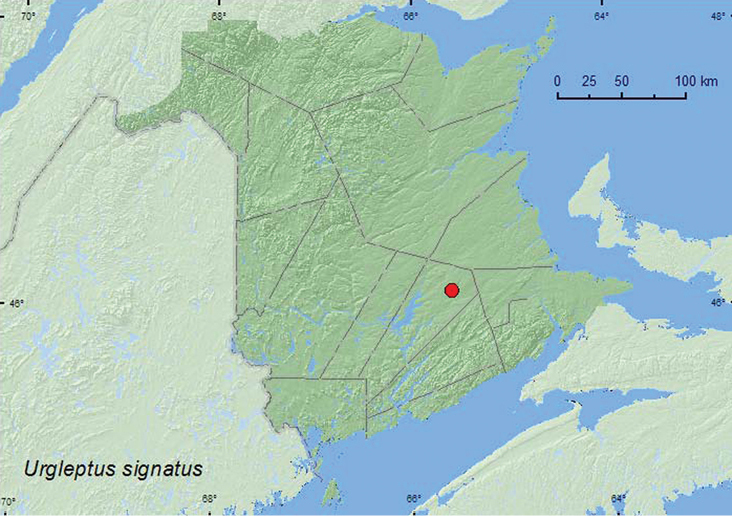
Collection localities in New Brunswick, Canada of *Urgleptus signatus*.

## Supplementary Material

XML Treatment for Neospondylis upiformis

XML Treatment for Acmaeops discoideus

XML Treatment for Phymatodes aereus

XML Treatment for Phymatodes maculicollis

XML Treatment for Phymatodes species

XML Treatment for Phymatodes testaceus

XML Treatment for Sarosesthes fulminans

XML Treatment for Anelaphus villosus

XML Treatment for Urgleptus signatus
